# Kinematic Analysis During Straight Line Free Swimming in Horses: Part 2 - Hindlimbs

**DOI:** 10.3389/fvets.2021.761500

**Published:** 2022-01-31

**Authors:** Emma Santosuosso, Renaud Leguillette, Tatiana Vinardell, Silvio Filho, Shannon Massie, Persephone McCrae, Sarah Johnson, Campbell Rolian, Florent David

**Affiliations:** ^1^Faculty of Veterinary Medicine, University of Calgary, Calgary, AB, Canada; ^2^Equine Veterinary Medical Center, Member of Qatar Foundation, Doha, Qatar; ^3^College of Health and Life Sciences, Hamad Bin Khalifa University, Member of Qatar Foundation, Doha, Qatar; ^4^Al Shaqab's Endurance Department, Member of Qatar Foundation, Doha, Qatar

**Keywords:** swimming, kinematics, joint, flexion, extension, angular velocity, range of motion (ROM), rehabilitation

## Abstract

**Background:**

Swimming is used for rehabilitation and conditioning purposes in equine sports medicine. We described the swimming kinematics of the equine forelimbs in Part 1. The aim of Part 2 is to assess stifle, tarsus, and hind fetlock joints kinematics in swimming horses. The objectives were 1- to calculate and compare joint angles during swimming against passive mobilizations (PM), 2- to determine joints angular velocities during a swimming stride cycle.

**Methods:**

Eleven elite endurance horses were used to swim in a 100-meter straight pool. Underwater (swimming) and overground PM videos were recorded from the horses' left side. Joint markers were applied on the lateral hoof wall, lateral metatarsal epicondyle, lateral aspect of the talus, lateral femoral epicondyle, and great trochanter of the femur. As a reference, maximal fetlock, tarsus, and stifle flexion/extension angles were determined during PM overground. Differences between angle extrema, angular velocities, and range of motion (ROM) were statistically compared.

**Results:**

The tarsus ROM was similar during PM and swimming. The stifle and fetlock ROM were greater during PM, although the stifle flexion was greater during swimming. The stifle and tarsus had the greatest hindlimb angular velocity during the swimming cycle. Greater angular velocities were observed during the retraction phase for all the hindlimb joints.

**Conclusion:**

A short retraction phase with great angular velocity for the joints of interest characterized the swimming pattern observed. Swimming may be beneficial in horses when an increased ROM of the tarsus and stifle or a reduced fetlock extension is indicated for rehabilitation purposes.

## Introduction

Historically, swimming has been used in racehorses for conditioning and rehabilitation purposes (1, 2). Previous studies have investigated the cardiovascular (3, 4), muscular (5, 6), and respiratory (7, 8) responses in horses during swimming. In a companion manuscript, forelimb kinematic data of swimming horses were described (9). This study reports the kinematics of the hindlimb during swimming, where it is thought to have a propulsive role (10).

Research conducted on water treadmills has shown kinematic changes of the limbs and back in response to the depth of water (11). These changes included an increased range of motion (ROM) in several joints, reduced segmental accelerations, and increased angular velocities (12–14), indicating the potential therapeutic benefits of aquatic therapy (15). Despite the empirical use of swimming for the rehabilitation of horses, concerns have been raised on the potential risks associated with the excessive extension of the spine and limbs (16). A description of the kinematics of the equine hindlimb would provide crucial information to better understand the swimming pattern and motion used by horses, therefore, contributing to the optimization of swimming rehabilitation protocols.

Swimming kinematics have previously been studied in other species including dogs (17), turtles (18), and aquatic mammals (19, 20). In swimming dogs, the limb strokes were of greater amplitude and displayed increased stifle extension compared to walking or trotting overground (21). The general term “dog paddle” has been used to characterize the swimming motions of several species including muskrats, mice, and dogs (17). The main objective of a swimming organism is to produce enough forward force (thrust) to overcome the induced resistive force (drag), acting parallel to the direction of motion, and balance any lateral and vertical forces to avoid sinking (22). Improvement in speed, thrust production, and efficiency has been accomplished by a change of swimming mode in the animal kingdom (10). Terrestrial and semiaquatic mammals employ a drag-based propulsion with paddling appendages, whereas fully aquatic mammals use a lift-based propulsion with oscillating hydrofoils (10).

The purpose of this study was to assess the hindlimb kinematics of horses during swimming. The specific objectives of the study were: 1- to compare hindlimb joint angles and ROM during swimming with those obtained during passive mobilization (PM) (used as a reference), 2- to obtain and compare angular velocities during the protraction and retraction phases of the swimming stride. We hypothesized that any joint ROM would be greater during PM than during swimming, that the angular velocity will differ between joints (with greater values obtained for more distal joints), and those absolute values of angular velocity are greater during retraction than protraction.

## Materials and Methods

The study was approved by the Institutional Animal Care and Use Committee of the Equine Veterinary Medical Center, a member of Qatar Foundation (EVMC-2020-1135) and performed at the Al Shaqab Equine Exercise Center where the straight pool is located.

### Horses

Eleven elite Arabian endurance horses (7 geldings and 4 mares; mean age ± SD = 13.8 ± 3.2 years old; weighing 427 ± 41.1 kg) were enrolled in the study. Each horse was confirmed to be free of lameness based on history and a detailed clinical examination. All the horses had previously undergone a minimum of two months of swimming training and were acclimated to the pool and distance used for the study.

### Experimental Protocol

The experimental protocol was similar to the one previously utilized in the companion study on the forelimb (9). Data were collected in an indoor, 100 m long, 2.95 m wide, and 3 m deep, straight pool that allowed free swimming in a straight line over at least a 70 m distance. The pool water was transparent and lit from both above and underwater. Swimming speed was recorded, and horses were allowed to swim at their preferred speed without interference from the handler.

Videos were recorded from the left side of the horse, with cameras placed 25 meters away from the swimming start zone to ensure that horses had achieved a steady swimming pattern. The two-dimensional (2D) movements were recorded using two underwater digital video cameras^1^ with an acquisition rate of 60 frames/s and a resolution of 1,440 pixels. The cameras were positioned by a diver on the left wall of the pool using suction cups at approximately 50 cm under the water surface and at an exact horizontal level (confirmed with a level). The two cameras were set at a 2 m distance from each other. Before each recording, a calibration ruler was placed in front of the field-of-view of each camera at the same distance as the left hindlimb would be during swimming. The handlers were instructed to gently guide the horses so that they would swim closer to the far wall of the pool (relative to the cameras) to ensure proper framing of the entire legs of the horses. All the horses swam two lengths, the first one being a warm-up length, and the second one being when the strides were recorded; the recordings were acquired on one swimming pass only.

**Figure 1:**
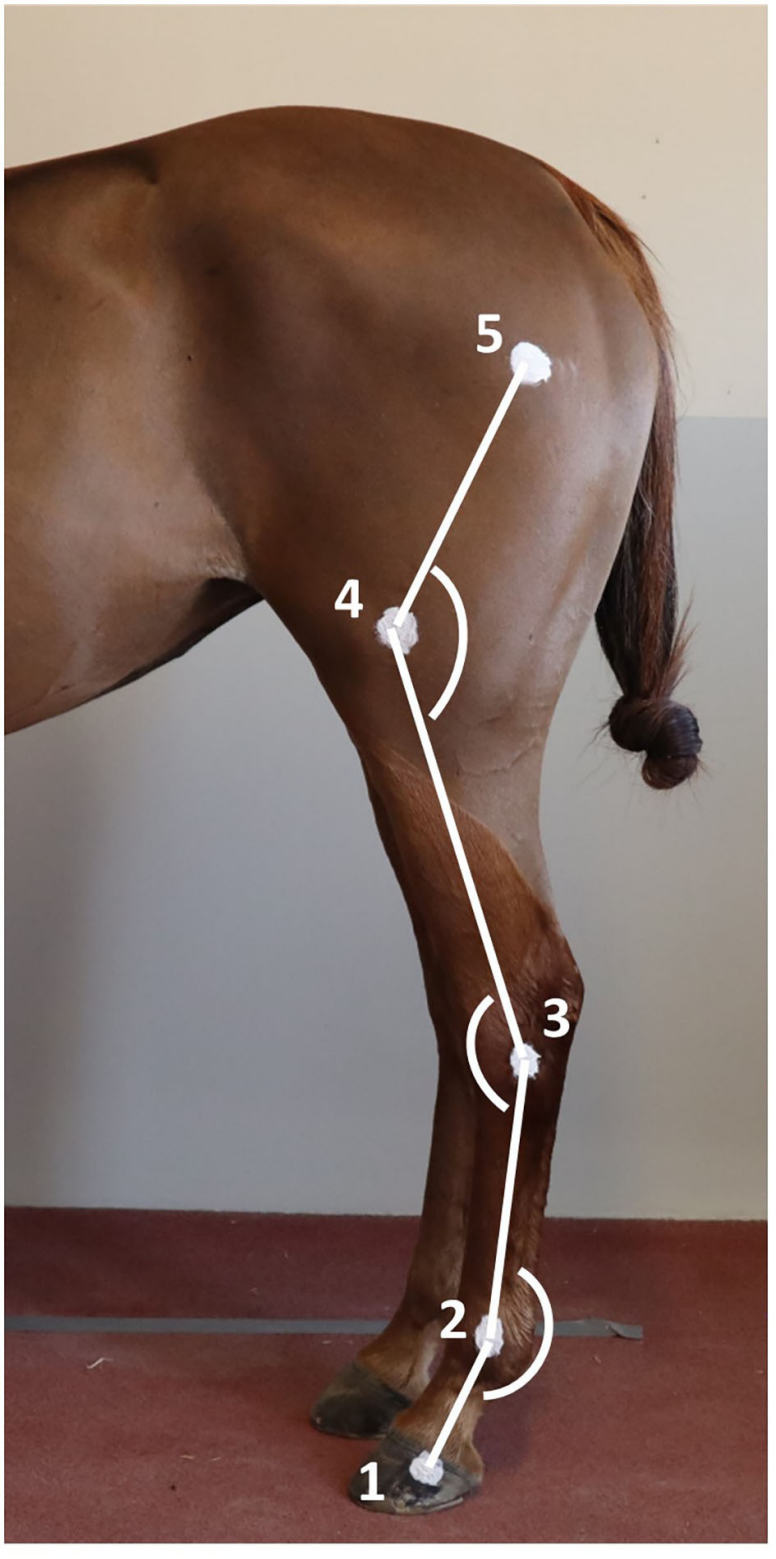
Position of the markers on the hindlimb anatomical landmarks and angles measured. 1) Lateral hoof wall; 2) Lateral metatarsal epicondyle; 3) Lateral tubercle of the talus; 4) Lateral femoral epicondyle; and 5) Great trochanter of the femur.

Two-centimeter diameter zinc oxide cream^2^ round markers (13, 23) were applied on the left hindlimb at the level of 1- hoof wall (foot divided into 2 parallel halves and marker placed 1 cm distal to the coronary band, on the hoof quarter, approximately superimposed with the plantar aspect of distal interphalangeal joint), 2- lateral metatarsal epicondyle, 3- lateral tubercle of the talus (immediately proximal and dorsal to the 4^th^ tarsal bone), 4- lateral femoral epicondyle, and 5- great trochanter of the femur (Figure 1) (24). The fetlock, tarsus, and stifle joint angles were respectively defined as the plantar angle between segment joining markers 1, 2, and 3; dorsal/cranial angle between segment joining markers 2, 3, and 4; and, the caudal angle between segment joining markers 3, 4, and 5 (Figures 1, **3**). The ROM was determined as the difference between maximal flexion and extension of each joint.

**Figure 2:**
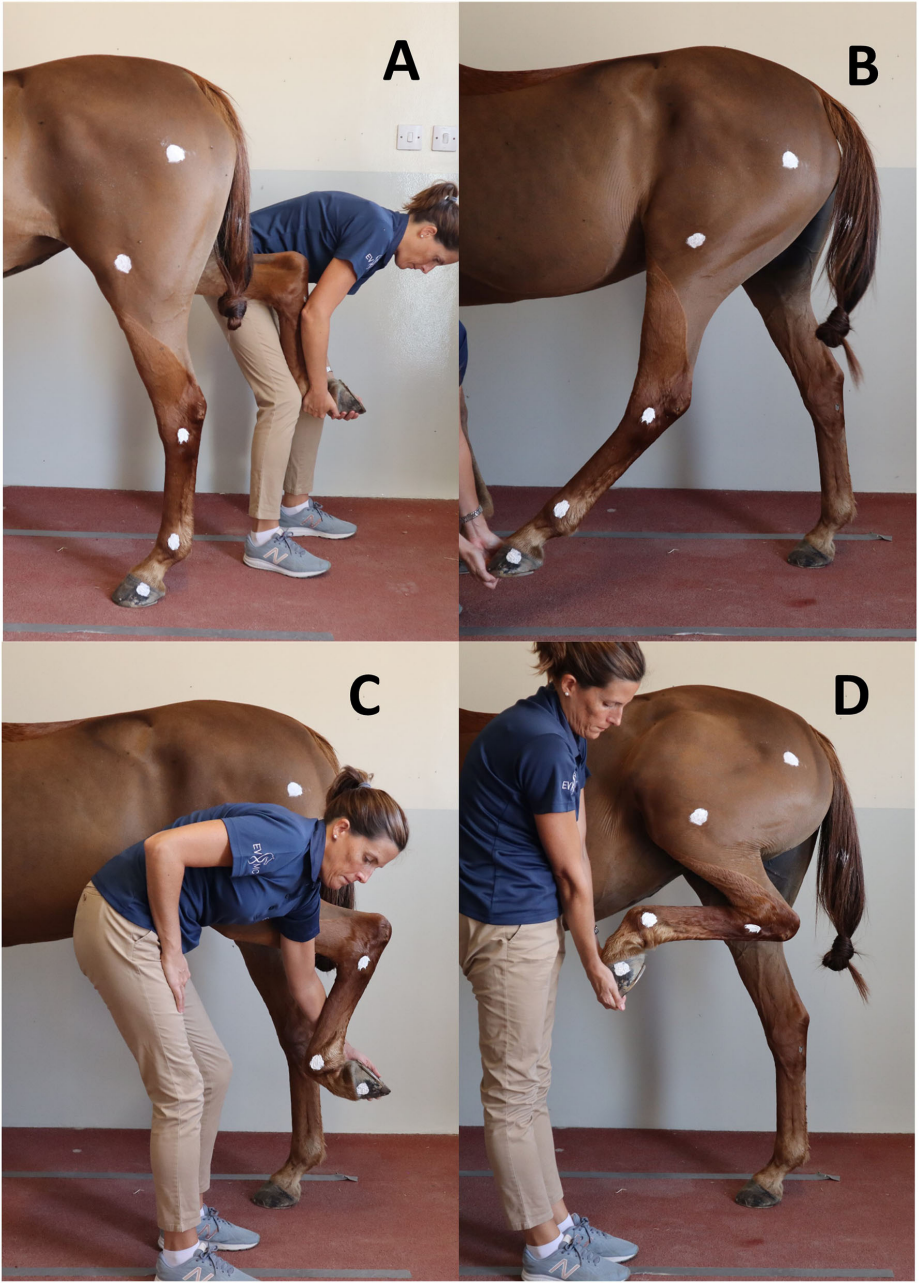
Positions obtained for maximal flexion and extension of the hindleg during passive mobilization. **(A)** Maximal fetlock and tarsus extension; **(B)** Maximal stifle extension; **(C)** Maximal fetlock flexion; **(D)** Maximal tarsus and stifle flexion.

To establish a reference to compare to swimming, maximal PM for the joints of interest was recorded for each horse in a static position overground and ROM were calculated (Figure 2). Two handlers were trained to ensure standardization of maximal passive joint flexion and extension, defined as the lowest (flexion) and greatest (extension) measured angles for a given joint. Horses stood over an imaginary rectangular frame drawn on the ground to ensure the body of the horse would stay parallel to a video camera^3^ set on a tripod placed 2 m from the left side of the horse while PM were recorded. The height of the camera on the tripod was 118 cm. For the extension of the fetlock and the tarsus, the joint angles were collected while the horse was bearing full weight on the left hindleg while standing in a vertical position (Figure 2A). For the extension of the stifle, the hindlimb was mildly elevated and the leg protracted until maximal stifle extension was achieved (Figure 2B). For the fetlock flexion, the hindlimb was grasped by the toe, kept mildly elevated and the digit was flexed until maximal fetlock flexion was achieved (Figure 2C). For the tarsus and stifle flexion, the hindlimb was grasped by the toe and the handler proceeded to lift until maximal flexion of the tarsus and stifle were achieved (Figure 2D). Maximal PM flexion and extension for a given joint were defined as the ultimate physiological joint movements possibly obtained by a handler safely and without inducing discomfort to the horse or a loss of balance. The entire procedure was repeated three times to assess intra- and interoperator variability. The order of the joints assessed was randomized.

### Kinematic Analysis of Swimming

**Figure 3:**
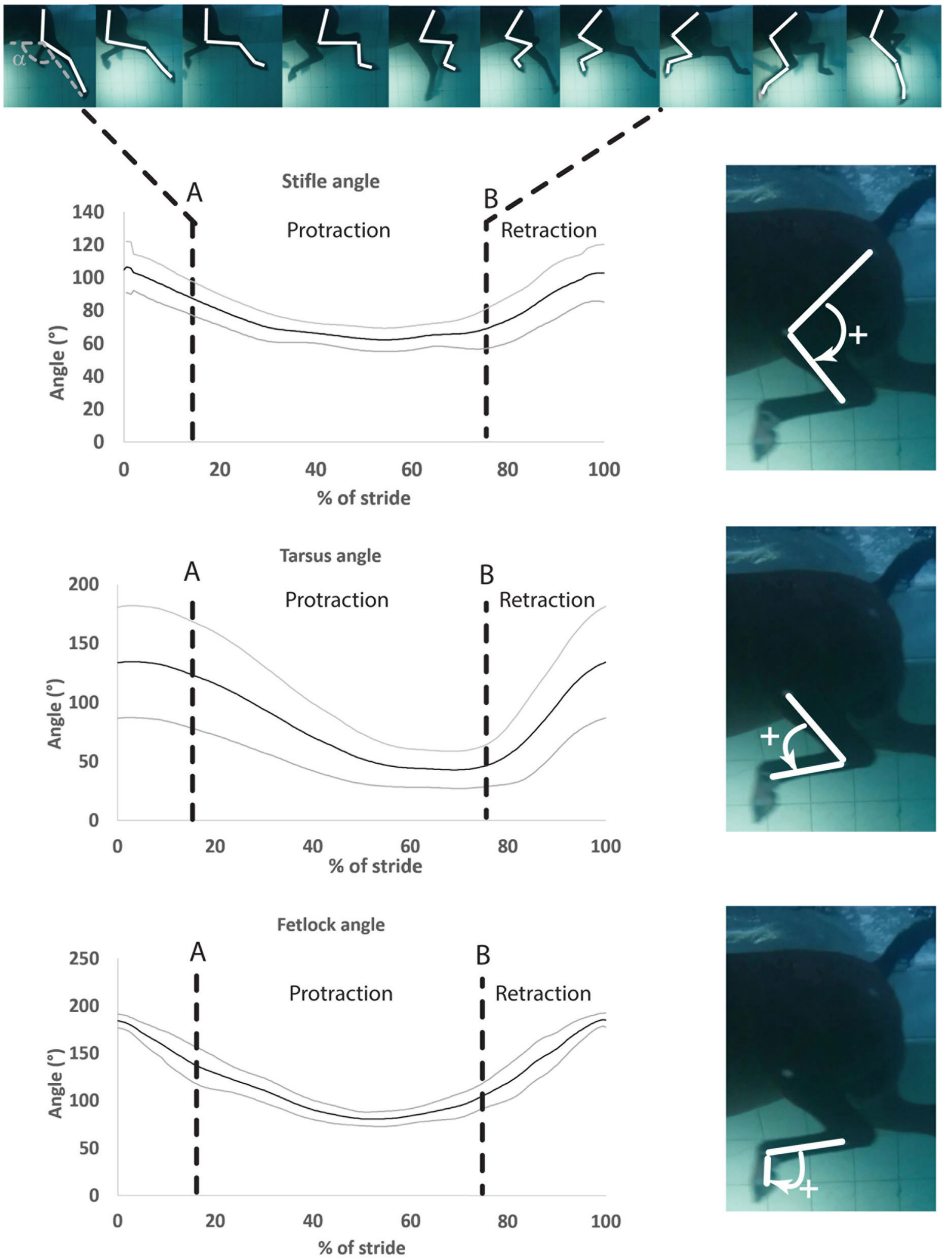
Mean ± SD of the stifle, hock, and fetlock joint angles during a complete swimming stride (protraction and retraction). A, Start of protraction phase; B, Start of retraction phase; α, protraction–retraction angle. The arrow indicates the flexion of the joint of interest on the side illustration.

One swimming stride was defined as the duration between two consecutive instances of maximum fetlock extension. Therefore, the onset of the stride was defined as the instant when the fetlock angle was maximal (Figure 3). Videos from each camera were analyzed independently. Specific swimming speed during the recordings was derived from the distance the horse (tarsus marker) covered during the time, as determined by the frame rate. The videos from both cameras were reviewed for quality and only those with clear visibility of all the skin markers were used for analysis. Using the center of the markers, manual marker tracking was carried out by using a 2D motion analysis software^4^ (25). The 2D coordinates obtained in the X- and Y-axes were exported as a function of time to a spreadsheet. Manual interpolation was performed to normalize data and to express time as a percentage of total stride duration. Angle vs. time profiles (as a percentage of the stride) were obtained for the three joints (Figure 3).

For the joint angle interpretation, the bigger the extension angle or ROM, the more important extension or ROM for a given joint. For flexion, it was the opposite, and the smaller the flexion angle, the more important the flexion for a given joint.

Angular velocity was calculated using the first derivative of the angular displacement (Equation 1):


$$\begin{array}{l} \;\omega =\frac{\Delta \theta }{\Delta t}\\ \;Equation\;1:\;Angular\;velocity\;equation\\ \omega :\;Angular\;velocity;\Delta \theta :\;Angle\;difference;\Delta t:\;Time\;difference. \end{array}$$

They were assigned a positive sign when the angle difference was positive (extension of the joint) and negative otherwise (flexion of the joint). The sign provided information on the direction of the joint motion and the absolute value provided information about how fast this motion occurred. Angular velocity spreadsheets were exported into a computing software^5^ and a local regression (LOESS function) was applied with a constant span (26). The maxima/minima angles, ROM, along with the maxima (maximum angular velocity during extension of the joint) and minima (maximal angular velocity during flexion of the joint) of angular velocities were normalized over the duration of a stride to enable comparisons among horses. Mean angular velocities for all the angles were calculated during the limb protraction and retraction phases of the stride.

### Kinematic Analysis of Passive Mobilization

For each horse, six briefs (3 to 4 s) videos were imported into the 2D motion analysis software^4^ and the mean of the maximal flexion and extension joint angles were obtained with the two operators. The ratios of maximal flexion, extension, and ROM obtained during swimming to those obtained during PM were calculated. For extension and ROM, ratios greater than 1 indicated that extension or ROM for a given joint were greater during swimming, while ratios smaller than 1 indicated they were greater during PM. For flexion, it was the opposite, and ratios greater than 1 indicated that flexion for a given joint was greater during PM, while ratios smaller than 1 indicated flexion was greater during swimming.

When significant variations between flexion/extension or ROM during swimming and PM were noted for a given joint, the difference in percentage was calculated and expressed as the difference between them divided by their average multiplied by 100 (percentage difference formula = [(a-b)/((a+b)/2)] × 100, where a and b are the two angles/numbers compared).

### Statistical Analysis

A Shapiro-Wilk test was performed to test the assumptions of normality. A one-way repeated measures ANOVA followed by a Tukey *post-hoc* test was performed to compare the differences between minimal and maximal angles, ROM, and minimal and maximal peak angular velocities of each joint. Values obtained during PM and swimming were compared using a one-way ANOVA. Mean angular velocities obtained during protraction were compared with the values obtained during retraction using a paired *t*-test. Statistical significance was set at *p* < 0.05. All results are presented as mean ± SD relative to the number of horses (mean ± SD relative to the number of available strides calculated first for each horse), as datasets were normally distributed. Inter-observer agreement was assessed using a Bland-Altman test (limit of agreement: 95% CI).

## Results

### Swimming Stride Parameters

A range of 1 to 4 strides were initially video recorded for each of the eleven horses, for a total of 21 strides. Two strides were excluded from the analysis for the tarsus and fetlock joints and nine of these strides were excluded from the analysis for the stifle joint because of the inability to consistently visualize all the markers. A total of 19 full strides were kept for analysis for both fetlock and tarsus joints from 9 horses (2 horses with 1 stride, 5 horses with 2 strides, 1 horse with 3 strides, and 1 horse with 4 strides). A total of 12 strides were analyzed for the stifle joint from 8 horses (4 horses with 1 stride and 4 horses with 2 strides). All the swimming stride parameters are presented in Table 1.

**Table 1:** Hindlimb stride parameters in horses during free (untethered) swimming (*n* = 11).

**Parameters**	**Mean ± SD**
Speed (m/s)	1.1 ± 0.2
Cycle duration (s)	1.2 ± 0.3
Protraction phase (s)	0.8 ± 0.2
Retraction phase (s)	0.4 ± 0.08
Swimming stroke length (m)	1.2 ± 0.3

### Joint Angles

Angle-time (as a percentage of the stride) diagrams obtained for the fetlock, tarsus, and stifle joints are provided in Figure 3. Maximal flexion was first reached by the stifle (mid protraction), followed by the fetlock (mid protraction), and finally by the tarsus (late protraction) during the protraction phase, as shown in Figure 3 and Table 2. The maximal extension was first reached by the tarsus (mid retraction), rapidly followed by the fetlock and stifle (also mid retraction) during the retraction phase of the swimming stride (Figure 3; Table 2). The joint with the greatest flexion was the tarsus (*p* = 0.0001), while the joint with the least flexion was the fetlock. The joint with the greatest extension and ROM was the fetlock (*p* < 0.0001 for both), while the joint with the least extension and ROM was the stifle.

**Table 2:** Mean ± SD joint angle parameters in horses during free swimming (n = 9 for tarsus/fetlock; n = 8 for stifle).

	**Stifle**	**Tarsus**	**Fetlock**
**Angles**	
Maximal extension (°)	111.5 ± 17.1^a, b^	154.7 ± 4.1^a, c^	188.9 ± 5.98^b, c^
% stride at max extension	98.8 ± 11.7	86.6 ± 30.5	98.4 ± 4.6
Maximal flexion (°)	60.5 ± 8.3^d, e^	43.7 ± 5.1^d, f^	76.8 ± 5.7^e, f^
% stride at max flexion	52.4 ± 10.6	64.3 ± 3.3	54.6 ± 4.9
Angular ROM (°)	50.9 ±17.3^g, h^	110.7 ± 4.8^g^	112.2 ± 10.1^h^
**Angular velocity**	
Maximal (positive) angular velocity (°/s)	270.0 ± 53^a^	650.0 ± 95^a^	430.0 ± 220
% stride at maximal (positive) angular velocity	90.1 ± 5.2	88.1 ± 4.6	86.3 ± 10.7
Maximal (negative) angular velocity (°/s)	–170.0 ± 128.9	–360.0 ± 110	–370.0 ± 160
% stride at maximal (negative) angular velocity	11.6 ± 5.2	35.6 ± 4.4	20.4 ± 7.7
Mean angular velocity during protraction (°/s)	–13.9 ± 6.7^a^	–100.7 ± 25.4^a, b^	–23.4 ± 16.9^b^
Mean angular velocity during retraction (°/s)	125.3 ± 72.4^a, c^	403.7 ± 64.3^a, b^	288.1 ± 64.8^b, c^

*Letters indicate significantly different values between joints (p < 0.05). Comparisons between stifle and tarsus or fetlock values are only for the 8 horses who had stifle values. Measured angles are presented in Figure 1*.

### Angular Velocity During Protraction and Retraction Phases

The absolute value of the angular velocities of the three joints was significantly greater during retraction than during protraction (*p* = 0.0011) (Table 2).

Maximal negative angular velocity during protraction was first achieved by the stifle, followed by the fetlock and tarsus joints while the maximal positive angular velocities were reached in the mid retraction phase for all the joints (Table 2).

Irrespective of the phase (retraction and protraction phases), the tarsus had the highest positive and negative angular velocity compared with other joints (*p* = 0.05).

### Comparison Swimming-Passive Mobilization

The Bland-Altman scatter plots revealed a good agreement between handlers during PM (Appendix 1). PM was performed on 9 of 11 horses as 2 of them were lost to follow-up. Mean joint flexion, extension, and ROM, as well as the ratios of swimming to PM values for each joint, are presented in Table 3. Maximal stifle flexion was approximately 28% greater during swimming (60.5 ± 8.27) than during PM (79.9 ± 12.7) (*p* = 0.008). In contrast, stifle extension was approximately 30% greater during PM (141.5 ± 11.5) than during swimming (104.2 ± 8.2) (*p* = 0.008). The ROM of the stifle and fetlock joints obtained during PM were significantly greater (+18% and +10%) than those obtained while swimming (*p* = 0.008). No significant difference was observed in the tarsus joint.

**Table 3:** Mean ± SD flexion, extension, and ROM comparisons obtained during passive mobilization (PM) and free swimming (n = 9 for tarsus/fetlock; n = 8 for stifle).

	**Stifle**	**Tarsus**	**Fetlock**
**Passive mobilization**			
Max flexion (°)	79.9 ± 12.7	44.9 ± 4.7	89.5 ± 7.1
Max extension (°)	141.4 ± 11.5	155.1 ± 6.2	212.7 ± 4.3
ROM (°)	60.9 ± 13.9	110.2 ± 6.5	123.7 ± 5.7
**Ratios swimming vs. Passive mobilization**			
Ratio flexion	0.7 ± 0.2 ^*^	1.0 ± 0.0	0.9 ± 0.1
Ratio extension	0.7 ± 0.3 ^*^	1.0 ± 0.0	0.9 ± 0.0^*^
Ratio ROM	0.7 ± 0.6^*^	1.0 ± 0.0	0.9 ± 0.1^*^

*Mean ± SD ratio indicates the ratio of maximal flexions (extension, ROM) obtained during swimming to maximal flexions (extension, ROM) obtained during passive mobilization.*

* *Indicates a statistically significant difference (p < 0.05) between the numerator and denominator of the ratio. For extension and ROM, a ratio greater than 1 indicates that extension or ROM for a given joint is greater during swimming, while ratios smaller than 1 indicates they were greater during PM. For flexion, it is the opposite and a ratio greater than 1 indicates that flexion for a given joint is greater during PM, while a ratio smaller than 1 indicates flexion was greater during swimming*.

## Discussion

This study is the first to describe equine hindlimb kinematics during swimming and complements the forelimb data reported in Part 1 (9). Significantly lower ROM of the stifle and fetlock were observed during swimming compared with PM. The greatest swimming ROM were reached by the tarsus and fetlock. The greatest absolute values of the angular velocity were observed during the retraction phase for all joints of interest, with the tarsus reaching the highest angular velocities of all.

### Joint Angles

Passive mobilizations were used as a reference to compare joint ROM during swimming. While both PM and swimming are used in rehabilitation programs (16), PM is a static exercise that does not involve the cardio-respiratory system or active muscle recruitment but requires the acceptance of the horse. Swimming and other aquatic therapies, such as water treadmill exercise, present the advantage of imposing a variable workload, which may be utilized to maintain or increase fitness (23).

The greater ROM of the fetlock observed during PM was likely due to an increased extension, which occurs during ground contact, forcing extension secondary to weight-bearing. If the fetlock joint extension is reduced during swimming, this could be an interesting feature of swimming exercise for rehabilitation purposes. The greater ROM of the stifle observed during PM was likely due to an increased extension induced by the operator when the leg was protracted maximally with the foot slightly elevated from the ground, mimicking what occurs during ground contact (27).

While overall stifle ROM was greater during PM than swimming, swimming induced a greater degree of stifle flexion. The tarsus also experienced a high degree of flexion during swimming, with no difference in ROM during swimming compared to PM. This finding is similar to previous observations in rats, where the extent of knee and ankle flexion was greater during swimming than during walking on a 10° slope (28). Gruner *et al*. hypothesized that such a hyperflexion during swimming would bring the limb closer to the body and lower its hydrodynamic resistance during protraction, decreasing the effective area exposed to drag and therefore improving mechanical efficiency (28).

The degree of joint flexion reported during swimming in this study is greater than what has previously been described at a walk-in Spanish horses (29) or ponies (30), and at a trot in various other horse breeds (27, 30, 31). In addition, the ROM of the stifle and tarsus were greater during swimming than what has previously been reported during the walk and trot (27, 29–31). These findings are in accordance with previous canine studies where hindlimb ROM of healthy dogs was greater during swimming than during walking (15, 28). As shown in dogs recovering from rupture of the cranial cruciate ligament (15), the increased ROM of the stifle observed during swimming may also be beneficial for the rehabilitation of various equine stifle injuries. For example, swimming, as an unloaded form of exercise, might be beneficial for horses suffering from menisci pathology or stifle joint osteoarthritis; horses suffering from the upward fixation of the patella may benefit from swimming exercise to rapidly build quadriceps muscle mass without reaching full stifle extension (fixation point); horses suffering from stifle fibrosis secondary to large periarticular hematoma/seroma may benefit from swimming to stretch the periarticular tissue and regain more rapidly a near-normal ROM. Future studies evaluating the benefits of swimming as a form of rehabilitation for the aforementioned conditions are warranted before any strong recommendations can be made.

Underwater treadmill exercise was also proven to modify kinematics of the walk in horses (13, 32). When comparing swimming to underwater treadmill exercise (13), the ROM of the hind fetlock for both aquatic exercises were similar (112.2° vs. 111°, respectively), although more (≈ +40%) fetlock flexion (76.8° vs. 110–130°, respectively) and less (≈ −20%) extension (188.9° vs. 220–240°, respectively) were noted during swimming. Based on this comparison, we can hypothesize that suspensory apparatus injuries may be better rehabilitated with swimming exercise in the early postinjury phase and later with underwater treadmill exercise, to take into consideration this increase in fetlock joint extension that would translate into increased load/stress on the recovering suspensory apparatus. For the tarsus, the ROM was substantially greater (110.7° vs. 60–70°, respectively) during swimming when compared with the underwater treadmill exercise (13) (≈ +70%) and this difference was largely because of an increased in tarsal flexion (43.7° vs. 100–105°, respectively) during swimming (≈ +60%). Based on this comparison, we can hypothesize that injured hocks with loss of flexion would be better rehabilitated first with an underwater treadmill and then with swimming exercise to aim at regaining a better ROM. No comparison is possible for the stifle joint at this stage as ROM, flexion/extension have not yet been reported to the knowledge of the authors during underwater treadmill exercise. Studies comparing both the aquatic exercises and assessing their therapeutic values on various types of injuries are warranted. They would be extremely valuable for veterinarians and physiotherapists to assist them in designing rehabilitation protocols that are likely to shorten the rehabilitation time in equine athletes.

### Angular Velocity

In dogs, along with greater ROM, greater angular velocities during swimming were observed (15). Such a motion enhancement was considered in dogs as an opportunity for rehabilitation of rupture of the cranial cruciate ligament (15). The buoyancy was hypothesized to reduce the mass of the limb in motion, therefore, facilitating greater angular velocities. A similar observation was made in horses on the water treadmill, where the angular velocity of the hindlimb increased compared with walking overground (33). Compared with the values reported during water treadmill exercise (33), greater angular velocities were obtained during swimming in this study. This indicates that faster limb movement is promoted during swimming compared to water treadmill exercise, without any loading of the joints. Swimming should also be considered as a substitution conditioning exercise to free paddock exercise in horses that are prone to self-injury in paddocks.

The duration of the retraction phase (when the limb is extending) was shorter than the protraction phase, counting for approximately 40% of the total swimming stride duration vs. 60% for the protraction (when the limb is flexing). This difference in phase duration was previously proposed as a phenomenon to increase thrust and decrease drag during swimming in muskrats (34), and therefore, contribute to a greater propulsive efficiency. This finding might apply to the horse as well. The locomotion of mammals is controlled by a neuromotor pattern that is highly adaptable. The main difference between swimming and moving overground is the absence of a rigid substrate that provides sensory information to modify muscle activation patterns. Consequently, during equine swimming, there is a shift in the kinematics that generates a propulsive force hydrodynamically by paddling.

The swimming pattern of the horses observed in this study was similar to the drag-based propulsion pattern previously described in other terrestrial mammals (10). In the drag-based propulsion model, terrestrial mammals display a vertical orientation of the limbs, and the forces required to overcome the drag is primarily generated by the hindlimbs (17). Assessing the hindlimb kinetics was beyond the scope of this study, however, such a model is also likely to be the case in swimming horses.

### Study Limitations and Opportunities for Future Studies

Due to the challenges associated with data collection underwater, a 2D analysis approach was utilized. However, this method fails to take into account the mediolateral motion of the limb and rotation of the joints and spine. Whether the rotation of the hindquarters is significant and contributes to a tracking overestimation or underestimation by positioning the hindleg in a non-perpendicular plane to the camera is currently unknown. Therefore, future studies are necessary to assess limb kinematics in three dimensions, especially the rotation of the trunk and hip.

In addition, despite the fact horses swam in straight lines, the entire recording could not be perpendicular to the camera. Actually, when horses just entered or exited the field-of-view of the camera, they were not completely perpendicular to the camera. This has possibly introduced a certain degree of unknown error in the angle measurements.

As shown in Figure 1, the most distal anatomic marker was located on the hoof capsule, close to the coronary band. The fetlock joint angle should therefore be better described as the digital joint angle. Anatomically, this point is likely to be close to the projection of the center of rotation of the distal interphalangeal joint. This indicates flexion and extension of the distal interphalangeal joint were likely to have minimal impact on the measured fetlock (or digital) joint angle. During contact exercise, flexion/extension of the proximal interphalangeal joint was reported to be 13 +/– 4 degrees at the walk and 14 +/– 4 degrees at a trot by Clayton *et al*. (35). The ROM of this joint is likely to be reduced during noncontact exercise such as swimming, giving an order of magnitude of the potential over or underestimation of the fetlock joint angle measured in this study.

The number of swimming strides used for kinematic analysis was relatively low in our study (1–4 strides per horse). This was due to the relatively narrow width of the pool and to the limited field-of-view of the cameras. Taken together, this made the chance of recording a complete swimming stride where all the anatomic markers could be clearly visualized low at each swimming passage. For further studies, more swimming passages should be included with 5–10 strides per horse ideally recorded to decrease variability. In this study, it should be noted that 2 horses represented approximately one third of the available strides for the fetlock and tarsus, which might have biased the analysis. A constant number of strides analyzed per horse would have been ideal and limited the influence of some horses in the overall result. We chose to include all the analyzable strides but to minimize the impact of some horses with more strides analyzable than others, as all the results were expressed as mean (relative to the total number of horses) of the individual means (relative to the total number of strides per horse) and displayed as such in Tables 2, 3.

## Conclusion

Like the other terrestrial mammals, horses display a drag-based propulsion swimming pattern. The greatest ROM was achieved by the tarsus and fetlock, primarily because of the increased flexion during swimming, while the stifle ROM was reduced during swimming because of the reduced stifle extension. The retraction phase was associated with the greatest angular velocities. Further studies assessing the back and limb kinematics during swimming are required. While the benefits of swimming are empirically recognized, especially for neurological (2) and orthopedic conditions (15) in both horses and dogs, future studies are still necessary to provide evidence for the implementation of swimming in the rehabilitation programs.

## Data Availability Statement

The raw data supporting the conclusions of this article will be made available by the authors, without undue reservation.

## Ethics Statement

The animal study was reviewed and approved by the Institutional Animal Care and Use Committee of the Equine Veterinary Medical Center, Member of Qatar Foundation, Doha, Qatar, under the protocol number EVMC-2020-1135. Written informed consent was obtained from the owners for the participation of their animals in this study. Written informed consent was obtained from the individual(s) for the publication of any potentially identifiable images or data included in this article.

## Author Contributions

ES, RL, SM, PM, TV, CR, and FD: hypothesis generation, experimental design, organizing and conducting the experiments, interpreting, analyzing the results, writing, and revising the manuscript. SF: experimental design and revising the manuscript. SJ: revising the manuscript. All authors contributed to the article, organizing and conducting the experiments, and approved the submitted version.

## Funding

This work was supported by the Equine Veterinary Medical Center, Member of Qatar Foundation - Intramural grant program (No. RG19_FD1) to FD and Faculty of Veterinary Medicine, Chair in Equine Sports Medicine, University of Calgary to RL.

## Conflict of Interest

The authors declare that the research was conducted in the absence of any commercial or financial relationships that could be construed as a potential conflict of interest.

## Publisher's Note

All claims expressed in this article are solely those of the authors and do not necessarily represent those of their affiliated organizations, or those of the publisher, the editors and the reviewers. Any product that may be evaluated in this article, or claim that may be made by its manufacturer, is not guaranteed or endorsed by the publisher.

## Footnotes

^1^GoPro Hero7 Silver, GoPro Inc., USA.

^2^Sudocrem, Forest Tosara LTD, Ireland.

^3^Canon EOS M50.

^4^KINOVEA version 0.9.1. Available for download at: http://www.kinovea.org

^5^R version 3.5.2b.
