# Supplementary figures and images for: Kinematic Analysis During Straight Line Free Swimming in Horses: Part 2 - Hindlimbs
Source: Front Vet Sci. 2022 Jan 31;8:761500. doi: 10.3389/fvets.2021.761500 (PMC8843043; doi:10.3389/fvets.2021.761500)

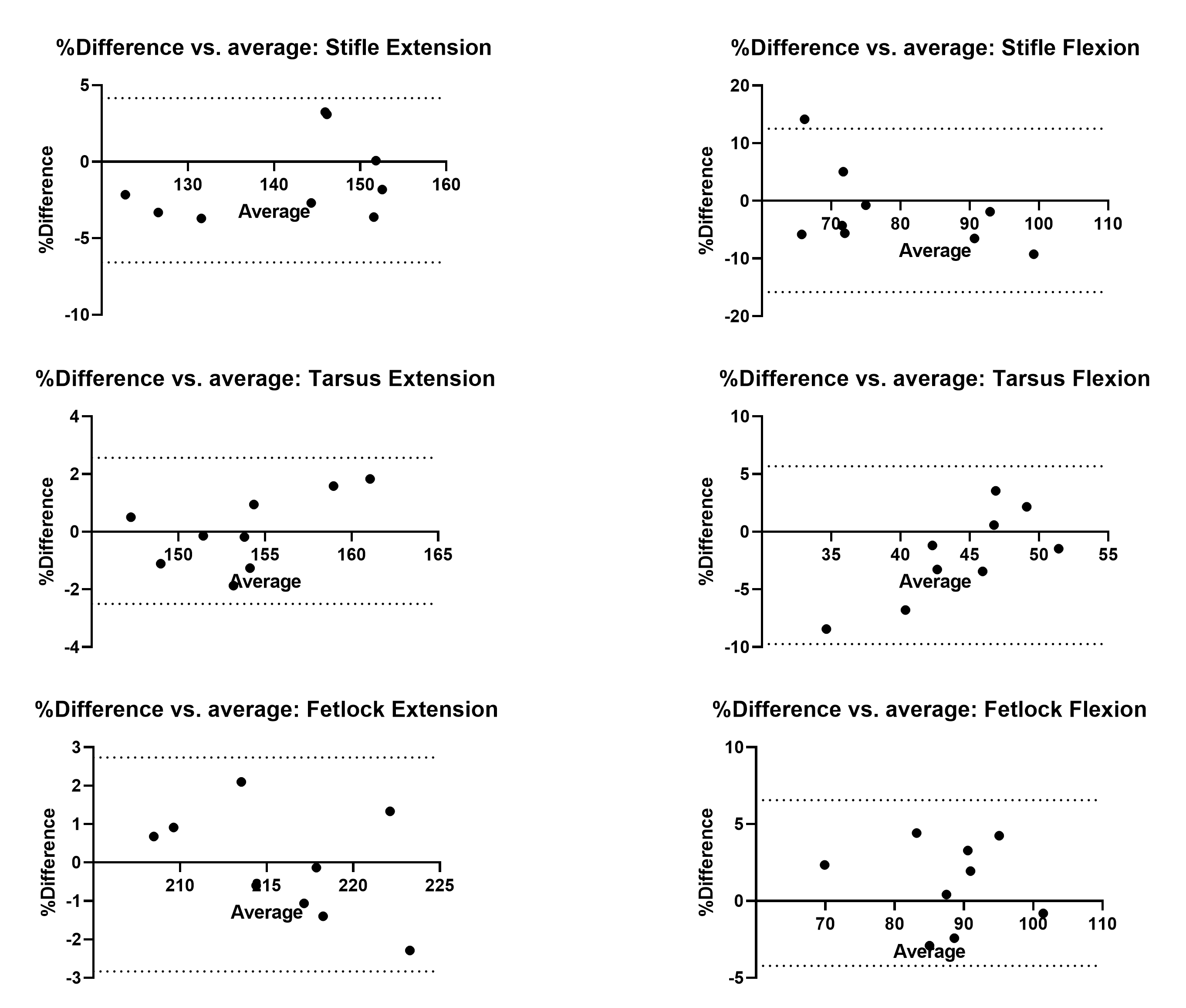

Supplement: Supplementary file 1 [file Image_1.TIF]
